# Validation and Practical Applications of Performance in a 6-Min Rowing Test in the Danish Armed Forces

**DOI:** 10.3390/ijerph18041395

**Published:** 2021-02-03

**Authors:** Oliver Funch, Henriette A. Hasselstrøm, Thomas P. Gunnarsson

**Affiliations:** 1 Department of Nutrition, Exercise and Sports, Faculty of Science, University of Copenhagen, 2200 Copenhagen, Denmark; wtc149@alumni.ku.dk; 2 Centre for Military Physical Training, Danish Defence Medical Command, 2100 Copenhagen, Denmark; fsk-c-mks02@mil.dk

**Keywords:** basic physical fitness test, 6-min rowing test, Cooper’s test, military personnel, re-testing, fitness test, physical performance

## Abstract

Personnel of the Danish Armed Forces must complete a yearly basic physical fitness test consisting of a Cooper’s 12-min run test (CRT) and four strength-related bodyweight exercises. However, there is no validated alternative to the CRT allowing injured or sailing personnel to conduct the yearly basic physical fitness test. Therefore, the aim of this study was to validate performance in a 6-min rowing ergometer test (6MRT) against CRT performance. Thirty-one individuals (M/F: 20/11, age: 34 ± 12 years) employed at the Danish Armed Forces completed testing on two independent days; (I) the CRT on an outdoor track and (II) a 6MRT with pulmonary measurements of breath-by-breath oxygen uptake. In addition, 5 participants (M/F: 4/1, age: 40 ± 10 years) completed re-testing of the 6MRT. No difference was observed between VO_2max_ estimated from the CRT and measured during the 6MRT. Absolute VO_2max_ correlated strongly (r = 0.95; *p* < 0.001) to performance in the 6MRT, and moderately (r = 0.80; *p* < 0.001) to performance in the CRT. Bodyweight (BW) and fat free mass (FFM) correlated stronger to performance in the 6MRT compared to the CRT. 6MRT re-testing yielded similar performance results. The 6MRT is a valid and reliable alternative to the CRT, allowing injured or sailing personnel of the Danish Armed Forces to complete the basic physical fitness test as required, albeit 6MRT performance demands must be made relative to bodyweight.

## 1. Introduction

### 1.1. Preface

Being a soldier is a physically demanding job and physical fitness is an important element of military tasks and military readiness [1]. The required fitness level depends on the task in question and the environment in which it is performed [2]. In general, good all-round physical fitness and strength is essential to meet the physical demands on the battlefield [3]. To ensure the capability of the soldiers to meet these demands and solve their military tasks, the different nation’s armed forces have various kinds of physical fitness tests and protocols that soldiers must pass [4]. In Denmark, military personnel must successfully complete a yearly basic physical fitness test to fulfil the demands of employment at the Danish Armed Forces. The basic physical fitness test is a physical minimum requirement test and includes a Cooper’s 12-min run test (CRT) and four strength-related bodyweight exercises (Table 1). The requirements for the basic physical fitness tests are differentiated on age but not gender. Hence, the requirement for the CRT is 1800–2200 m depending on age. For injured or sailing personnel, incapable of completing the CRT, alternative tests are available such as a shuttle run test, an ergometer bike test, or an indoor rowing test. The indoor rowing test lacks validation against the primary test, which is the 12-min CRT, and is preferred as the back-up test for injured personnel, or sailing personnel/personnel with no access to a running track. In addition, there is a lack of the specific ergometer bikes needed for completion of the ergometer bike test, limiting availability of this test in the Danish Armed Forces. In contrast, the Concept 2 rowing ergometer is common equipment in gyms and onboard vessels of the Danish Armed Forces. However, the indoor rowing test has only been validated against self-reported CRT distances ([5] personal communication). Hence, the Danish Armed Forces requested a 6-min rowing ergometer test (6MRT) to be validated against supervised CRT performance to enable more injured and/or sailing personnel to conduct the basic physical fitness testing.

### 1.2. Background

The primary factor contributing to performance during running is the speed associated with lactate threshold [6]. The lactate threshold during running is composed by the maximum aerobic capacity (VO_2max_), the sustainable fraction of VO_2max_, and running economy [6,7]. The primary contributor to performance during a 2000-m rowing event is the power output (Watt) associated with VO_2max_ [8,9]. In running and rowing alike, performance is highly dependent on individual VO_2max_ [6,7,8,9]. Hence, in both disciplines, athletes have high relative (up to 85 mL·kg^−1^·min^−1^ in elite long distance runners [7]) and absolute (>6 L·min^−1^ in elite male heavyweight rowers [8]) VO_2max_ values. In rowing, a high bodyweight is less detrimental to exercise performance compared to running [8,9,10,11,12,13], but rather correlate positively with performance [8,9], as bodyweight correlates positively with absolute VO_2max_ in rowers [10].

Engagement of a large muscle mass is important for assessment of VO_2max_ [14], and in running and rowing, a variety of test protocols are used to assess VO_2max_ [8,9,15,16,17,18,19,20,21]. In running, VO_2max_-testing has been assessed using incremental tests in large heterogeneous populations [21] and in smaller homogenous populations in training intervention studies [15,20], proving a reliable and valid method for assessment of VO_2max_ in non-specific populations. Further, VO_2max_ can be estimated by the CRT [16], and the CRT estimated VO_2max_ has been validated against gold-standard VO_2max_ measurements in both men and women [17,18,19]. However, compared to VO_2max_ values obtained during an incremental bicycle ergometer test, the CRT is less valid [22], suggesting a within-discipline specificity of the CRT. In rowing, assessment of VO_2max_ is often conducted as an incremental test [8,9,23] or as a 6-min all-out test [23]. Previous studies report no significant difference in VO_2max_ obtained during either type of test in elite lightweight rowers [23], indicating that the 6MRT may be valid for indirect measurements of VO_2max_, however whether this holds true for personnel employed at the Danish Armed Forces is unknown_._

### 1.3. Aims

The aims of the present study were to validate whether VO_2max_ during a 6MRT was comparable to the estimated VO_2max_ of a supervised CRT among personnel at the Danish Armed Forces, and to establish minimum demands for mean power output (MPO) during the 6MRT for comparison with the demands (distance) of a supervised CRT. Furthermore, to investigate whether the demand for MPO during a 6MRT was bodyweight (BW) dependent, and whether the 6MRT was reproducible, as there may be technical difficulties related to ergometer rowing, because of inexperience with rowing, among personnel at the Danish Armed Forces.

## 2. Materials and Methods

### 2.1. Study Design

The study was a cross sectional study including male and female personnel in the Danish Armed Forces. The study was conducted at the Danish Medical Command, Center for Military Physical Training (CMT) in Copenhagen and consisted of two tests; a CRT and a 6MRT. Testing was interspersed by at least 72 h, with an average of 25 ± 20 days (mean ± SD) between tests.

### 2.2. Subjects

Thirty-five individuals (11 females, 24 males) employed at the Danish Armed Forces volunteered to participate in the study. Thirty-one individuals (11 females, 20 males) (Table 2) completed both tests. All subjects gave their informed consent for inclusion before they participated in the study. The study was conducted in accordance with the Declaration of Helsinki, and the protocol was approved by the Research Ethics Committee for the faculties of SCIENCE and HEALTH, University of Copenhagen, (504–0333/21–5000). Further, the protocol was approved by the Danish Defense Medical Command, Center for Military Physical Training.

### 2.3. Testing

The CRT was conducted prior to the 6MRT for all subjects. Subjects were informed not to conduct endurance or resistance training within 48 h before testing. In case of injury and/or illness, testing was postponed, or subjects were excluded from the study. Subjects were instructed to avoid caffeine 24 h prior to testing [24], and to maintain their normal lifestyle (dietary habits and exercise training) between tests. Before testing, weight was measured (SECA 712, SECA GmbH, Hamburg, Germany), and subjects were equipped with a chest heart rate monitor (Polar Team^2^ HR monitors, Polar Electro, Kempele, Finland). Afterwards, subjects were instructed in the use of the RPE-scale (Borg’s scale). To access reliability of the 6MRT, five subjects (1 female, 4 males) were re-tested in the 6MRT after at least 48 h of rest.

#### 2.3.1. Cooper’s 12-Min Run Test

Subjects reported at CMT in the morning or afternoon and were introduced to the test protocol. Subjects conducted a 10-min individual warm-up. Then, 2 min after the warm-up the CRT was conducted on a 400-m cinders track (gravel track). Time was monitored by an instructor (Magma Pro, Hanhart, Prague, Czech Republic) and time-splits were given in 400-m intervals and 6, 8, 10, 11, and 11.5 min was announced. During the CRT, subjects received verbal encouragement to achieve maximal effort. A whistle marked the end of the test. Subjects were instructed to run as far and fast as possible, and the distance of the last 400-m lap not completed at the end of the test, was measured with a calibrated measuring wheel (Nedo Lightweight Meausering Wheel Econo, Nedo GmbH, Dornstetten, Germany). Immediately after the test, subjects reported test-related RPE values.

#### 2.3.2. 6-Min Rowing Test

Subjects reported at CMT in the morning or afternoon and were introduced to the test protocol. Prior to the test, subjects underwent 4-site skinfold measurements (Harpenden Skinfold Caliber, Baty International, West Sussex, UK) to determine individual body fat percentage and FFM. The 6MRT was conducted on a rowing ergometer (Concept 2, Model D, PM5, Concept 2 Inc., Morrisville, VT, USA) with damper set at 5 of 10. Subjects conducted a 5-min warmup at an individual pace. Post warm-up, there was a 2-min break before start of the 6MRT. During the 6MRT, subjects were instructed to perform maximally, and subjects received verbal encouragement to achieve maximal effort. Time (mm:ss), distance (m), MPO (W), and power per stroke (W) was monitored on the Concept 2 PM5-computer. VO_2_ and RER were measured breath-by-breath on a calibrated gas analyzing system (Quark Cardio Pulmonary Exercise Test (CPET), COSMED, Rome, Italy). Immediately after the 6MRT, subjects reported test-related RPE values.

### 2.4. Anthropometrics

Thirty of the thirty-one subjects included completed a 4-site skinfold measurement prior to the 6MRT. The skinfold measurement was conducted on the right-hand side of the torso. The average of two skinfold measurements was calculated and converted to body fat percentage (%). If the second skinfold measurement deviated by ≥1 mm from the first, a third measurement was conducted. FFM was subsequently found by subtracting body fat mass (kg) from the bodyweight (kg).

### 2.5. Calculations

Estimated VO_2max_ from the CRT was based on Cooper’s formula: VO_2max_ (mL·kg^−1^·min^−1^) = (distance (miles)−0.3138)/0.0278 [16,17], which was converted from miles to meters: VO_2max_ (mL·kg^−1^·min^−1^) = ((distance (m)*0.02233)−11.3). During the 6MRT, VO_2max_ and RER_max_ were determined as the highest values achieved over a 30-s period.

### 2.6. Statistics

SigmaPlot (version 14.0, Systat Software Inc., San José, CA, USA) was used for statistical analysis. Student’s paired t-tests were used to compare performance between the CRT and 6MRT, and to evaluate changes in performance between the repeated 6MRTs. Normal distribution was assessed based on Shapiro Wilk’s test and Wilcoxon signed ranked test was performed to compare non-parametric data. A significance level of *p* < 0.05 was chosen. To assess correlations, Pearson’s correlation coefficient (r) was determined by use of regression analysis, with the strength of association regarded as weak (<0.71), moderate (0.71–0.87), or strong (>0.87).

## 3. Results

### 3.1. Maximal Oxygen Uptake

**Figure 1:**
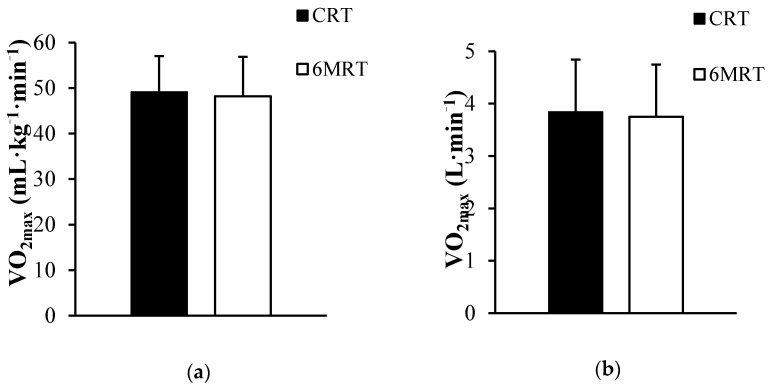
Data are presented as mean ± SD. Relative (**a**) and absolute (**b**) VO_2max_ estimated from the Cooper’s 12-min run test (CRT) and measured during the 6-min rowing test (6MRT) in 31 individuals employed at the Danish Armed Forces.

There was no difference between estimated VO_2max_ from the CRT and measured VO_2max_ during the 6MRT in relative (49.1 ± 7.9 vs. 48.2 ± 8.7 mL·kg^−1^·min^−1^) and absolute (3.8 ± 1.0 vs. 3.7 ± 1.0 L·min^−1^) values, respectively (Figure 1).

### 3.2. Comparison of Performance Characteristics Between Cooper’s 12-min Run Test and the 6-min Rowing Test

During the CRT, distance covered was higher (*p* < 0.05) compared to the 6MRT. Maximal and mean HR were higher (*p* < 0.001) and RPE was lower (*p* < 0.05) during the CRT compared to the 6MRT (Table 3).

### 3.3. Test Performance Correlations

**Figure 2:**
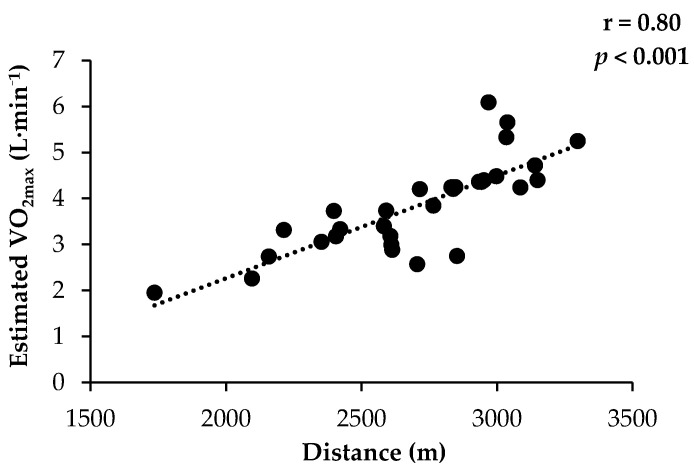
Correlation between distance covered and estimated absolute VO_2max_ calculated after Cooper’s 12-min run test (CRT) in 31 individuals employed at the Danish Armed Forces.

Distance covered during the CRT correlated moderately (r = 0.80, *p* < 0.001) to absolute VO_2max_ estimated from the CRT performance (Figure 2).

**Figure 3:**
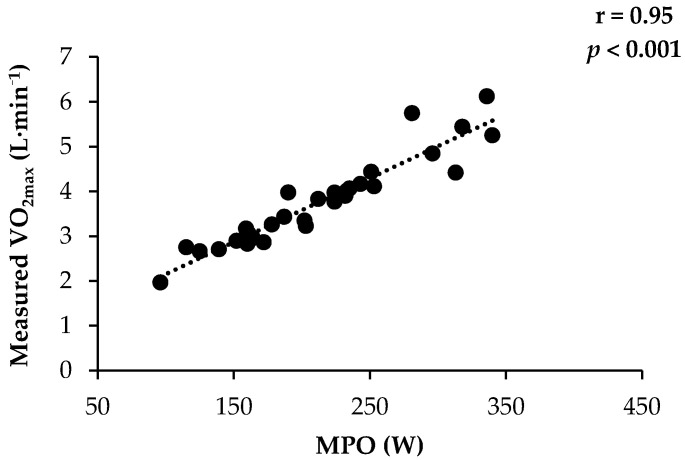
Correlation between mean power output and measured absolute VO_2max_ during the 6-min rowing test (6MRT) in 31 individuals employed at the Danish Armed Forces.

The MPO achieved during the 6MRT correlated strongly (r = 0.95, *p* < 0.001) to measured absolute VO_2max_ (Figure 3).

Bodyweight and fat free mass were stronger correlated to MPO during the 6MRT (BW: r = 0.73, *p* < 0.001; FFM: r = 0.87, *p* < 0.001) than distance covered during the CRT (BW: r = 0.30, *p* > 0.05; FFM: r = 0.55, *p* < 0.01) (Table 4).

### 3.4. Test-Retest Comparisons of the 6-min Rowing Test

Characteristics, performance, and physiological measures of five individuals (1 female, 4 male) performing 6MRT re-testing. There were no differences in characteristics, performance, and physiological measures between the first and second 6MRT (Table 5).

## 4. Discussions

The primary finding of this study was that VO_2max_ obtained during the 6MRT was similar to that estimated from the supervised CRT. Furthermore, mean power output during the 6MRT correlated stronger to absolute VO_2max_, bodyweight, and fat free mass than distance during the supervised CRT. Lastly, the 6MRT protocol was reliable with a high degree of reproducibility.

In the present study, VO_2max_ values obtained during the 6MRT did not differ from those estimated from the CRT. The estimated VO_2max_ from the CRT is validated in several studies [16,17,18,19], and this test is a well-known part of the basic physical fitness test of the Danish Armed Forces. The similarities in VO_2max_ values achieved between tests suggest that the 6MRT is a reliable and valid alternative to the CRT. Previous studies have failed to show a similarity in VO_2max_ obtained during treadmill running and ergometer rowing [25,26,27] or in VO_2max_ estimated from a CRT and measured during an incremental bicycle ergometer test [22]. However, the test protocols applied in these studies are different from the present study, mainly due to the application of incremental type tests in a non-military population [22,25,26,27]. Thus, they may not be applicable for direct comparison with the all-out test protocols in a military population utilized in the present study.

VO_2max_ is a well-known discriminator of endurance performance, and performance in the CRT correlated moderately with absolute VO_2max_, but weakly with fat free mass and bodyweight. However, there was a weak and non-significant positive correlation between bodyweight and running performance, which is in contrast to observations in previous studies showing a detrimental effect of increased bodyweight on distance running performance in well-trained runners [12] and top-100 world class track and field athletes [13]. Since the correlation between bodyweight and running performance was weak and non-significant, and the correlation between fat free mass and running performance was weak, neither measures were deemed as important performance indicators for the CRT. Performance in the 6MRT correlated stronger to absolute VO_2max_ compared to CRT performance. This observation is in accordance with previous studies in which strong correlations were found between absolute VO_2max_ and 2000 m rowing performance [8,9]. Bodyweight and fat free mass correlated moderately with performance in the 6MRT, which could be explained by a positive correlation between bodyweight and absolute VO_2max_ and fat free mass. One study demonstrated a positive correlation between absolute VO_2max_ and bodyweight in rowers [10], and given that muscle strength is vital for performance during a rowing event [28,29], this could explain the correlation between fat free mass and performance during the 6MRT. Consequently, it is crucial to take fat free mass, secondarily bodyweight, into account when establishing demands i.e., relative mean power output for performance in a 6MRT. For practical reasons based on everyday possibilities, a validated demand for mean power output during the 6MRT must be bodyweight dependent, as it is not possible to establish fat free mass during basic physical testing across the Danish Armed Forces.

Performance from 6MRT re-testing was not different from the first 6MRT, suggesting that the rowing protocol applied in the present study is reliable for assessment of VO_2max_ in the current population. Furthermore, ratings of perceived exertion and respiratory exchange ratio were similar between tests, suggesting a similar relative degree of fatigue. However, only five subjects completed re-testing (due to restrictions elicited by the COVID-19 pandemic), warranting caution on interpretation.

Heart rate during the 6MRT was significantly lower than during the CRT. This observation is in accordance with previous observations [25,26,27] and is most likely due to a greater muscle mass engaged during rowing, resulting in an improved venous return and therefore greater stroke volume and oxygen pulse during rowing compared to running. Thus, differences in heart rate during these tests do not necessarily reflect differences in exercise intensity, but rather differences related to exercise modality.

The higher ratings of perceived exertion after the 6MRT suggest that this test is harder to complete than the CRT. The majority of the Danish Armed Forces personnel are far more accustomed to running than rowing, which could be the underlying reason for the higher ratings of perceived exertion reported following the 6MRT compared to the CRT. Furthermore, the similar estimated and measured VO_2max_ values achieved implies a similar aerobic demand that is perceived harder during the most unfamiliar type of exercise modality. Lastly, the CRT is a well-known part of the basic physical fitness testing in the Danish Armed Forces, which could also explain the lower ratings of perceived exertion reported during this test compared to the 6MRT. Moreover, the lower heart rate and higher ratings of perceived exertion during the 6MRT could be explained by the greater demand for muscle strength, especially in the upper body, during rowing compared to running [28,29]. This could arguably cause greater peripheral muscular fatigue towards the end of the test, limiting the aerobic contribution during the event and inhibiting personnel to obtain maximal heart rate whilst causing greater perceived exertion compared to the CRT.

## 5. Conclusions

The present study shows that VO_2max_ values measured during the 6MRT are comparable to those estimated from the CRT in personnel of the Danish Armed Forces. Furthermore, the mean power output demands of the 6MRT must be made relative to bodyweight, due to the positive correlations between 6MRT performance and absolute VO_2max_, fat free mass and bodyweight. The 6MRT is considered a valid and reliable alternative to the CRT, and when taking bodyweight into consideration, minimum demands can be established for mean power output during the 6MRT that are comparable to the basic age-related demands for the CRT among personnel in the Danish Armed Forces. The rowing ergometer provides a safe, seated, and non-weight-bearing exercise type, which enable injured or sailing personnel of the Danish Armed Forces to complete the basic physical fitness test as required with minimum requirements comparable to the demands of the CRT.

## Figures and Tables

**Table 1:** Basic physical fitness test of the Danish Armed Forces.

	Endurance ^		Strength (Set × Repetitions)			
Age (Years)	Cooper’s Run Test (m)	Shuttle Run Test (Level)	Split Squats	Dips	Horizontal Pull Ups	Burpees
<49	2200	8.3	3 × 15	3 × 15	3 × 5	1 × 15
50–59	2000	6.9	3 × 12	3 × 12	3 × 3	1 × 10
>60	1800	5.9	3 × 10	3 × 10	3 × 2	1 × 5

^ It is sufficient to meet the minimum requirements of one endurance test when completing the basic physical fitness test in the Danish Armed Forces.

**Table 2:** Subject characteristics.

Male/female (*n*)	20/11
Age (years)	34.3 ± 12.0
Height (cm)	177.7 ± 8.5
BW (kg)	77.6 ± 12.1
FFM (kg)	60.0 ± 12.6
VO_2max_ (L·min^−1^)	3.7 ± 1.0
Estimated VO_2max_ (L·min^−1^)	3.8 ± 1.0

Data is presented as mean ± SD. BW: bodyweight, FFM: fat free mass, VO_2max_: maximal oxygen uptake measured during the 6-min rowing test, Estimated VO_2max_: maximal oxygen uptake estimated from the Cooper’s 12-min run test.

**Table 3:** Performance characteristics of a Cooper’s 12-min run test (CRT) and a 6-min rowing test (6MRT) in 31 individuals employed at the Danish Armed Forces.

	CRT	6MRT	*p*-Value
Distance (m)	2705 ± 356	1507 ± 160 *	*p* < 0.001
RPE (6–20)	17.2 ± 1.2	17.7 ± 1.3 *	*p* < 0.05
HR_mean_ (beats·min^−1^) ^§^	176 ± 10	166 ± 13 *	*p* < 0.001
HR_max_ (beats·min^−1^) ^§^	186 ± 10	176 ± 11 *	*p* < 0.001
MPO (watt)	N/A	211 ± 65	
RER_max_	N/A	1.08 ± 0.07	

Data are presented as mean ± SD. CRT: Cooper’s 12-min run test, 6MRT: 6-min rowing test, RPE: rating of perceived exertion, HR_mean_: mean heart rate, HR_max_: maximal heart rate, MPO: mean power output, RER_max_: maximal respiratory exchange ratio. ^§^: n = 27. * Different from CRT.

**Table 4:** Correlation between test performance of the Cooper’s 12-min run test (CRT) and a 6-min rowing test (6MRT), bodyweight and fat free mass in 31 individuals employed at the Danish Armed Forces.

	CRT (m)		6MRT (MPO, W)	
	r	*p*-Value	r	*p*-Value
BW (kg)	0.30 ^§^	*p* > 0.05	0.73 ^£^	*p* < 0.001
FFM (kg)	0.55 ^§^	*p* < 0.01	0.87 ^£^	*p* < 0.001

CRT: Cooper’s 12-min run test, 6MRT: 6-min rowing test, MPO: mean power output, VO_2max_: maximal oxygen uptake, BW: bodyweight, FFM: fat free mass. ^£^ Moderate correlation (r = 0.71–0.87). ^§^ Weak correlation (r < 0.71).

**Table 5:** Effects of re-testing in a 6-min rowing test (6MRT) in 5 individuals employed at the Danish Armed Forces.

	6MRT	Re-Test 6MRT	*p*-Value
	**Characteristics**		
Age (years)	40.0 ± 10.5		
Weight (kg)	77.2 ± 8.6	77.1 ± 8.7	0.75
FFM (kg)	59.1 ± 12.4	59.7 ± 12.3	0.27
	**Performance**		
Distance (m)	1498 ± 191	1501 ± 176	0.80
MPO (watt)	210 ± 77	210 ± 72	1.00
	**Physiological Measures**		
VO_2max_ (L·min^−1^)	3.9 ± 1.0	3.8 ± 1.0	0.20
VO_2max_ (mL·kg^−1^·min^−1^)	48.9 ± 9.0	48.3 ± 7.9	0.68
Exercise Economy (mL O_2_·watt^−1^)	19.2 ± 2.9	18.4 ± 1.7	0.25
RPE (6–20)	18.0 ± 1.6	18.0 ± 1.2	1.00
HR_mean_ (beats·min^−1^)	161 ± 13	161 ± 11	0.94
HR_max_ (beats·min^−1^)	172 ± 9	173 ± 12	0.52
RER_max_	1.07 ± 0.08	1.08 ± 0.08	0.84

Data are presented as mean ± SD. 6MRT: 6-min rowing test, FFM: fat free mass, MPO: mean power output, VO_2max_: maximal oxygen uptake, RPE: rating of perceived exertion, HR_mean_: mean heart rate, HR_max_: maximal heart rate, RER_max_: maximal respiratory exchange ratio.

## Data Availability

The data presented in this study are available on request from the corresponding author.
